# Relationship Between Polyunsaturated Fatty Acids and Psychopathology in the NEURAPRO Clinical Trial

**DOI:** 10.3389/fpsyt.2019.00393

**Published:** 2019-06-06

**Authors:** Maximus Berger, Barnaby Nelson, Connie Markulev, Hok Pan Yuen, Miriam R. Schäfer, Nilufar Mossaheb, Monika Schlögelhofer, Stefan Smesny, Ian B. Hickie, Gregor E. Berger, Eric Y. H. Chen, Lieuwe de Haan, Dorien H. Nieman, Merete Nordentoft, Anita Riecher-Rössler, Swapna Verma, Todd W. Mitchell, Barbara J. Meyer, Andrew Thompson, Alison Ruth Yung, Patrick D. McGorry, G. Paul Amminger

**Affiliations:** ^1^Orygen—The National Centre of Excellence in Youth Mental Health, Melbourne, VIC, Australia; ^2^Centre for Youth Mental Health, The University of Melbourne, Melbourne, VIC, Australia; ^3^Department of Psychiatry and Psychotherapy, Clinical Division of Social Psychiatry, Medical University Vienna, Vienna, Austria; ^4^Department of Child and Adolescent Psychiatry, Medical University Vienna, Vienna, Austria; ^5^Department of Psychiatry, University Hospital Jena, Jena, Germany; ^6^Brain and Mind Research Institute, University of Sydney, Sydney, NSW, Australia; ^7^Department of Child and Adolescent Psychiatry and Psychotherapy, University Hospital of Psychiatry Zurich, University of Zurich, Zurich, Switzerland; ^8^Department of Psychiatry, University of Hong Kong, Hong Kong, Hong Kong; ^9^Department of Psychiatry, Academic Medical Center, Amsterdam, Netherlands; ^10^Psychiatric Centre Bispebjerg, Copenhagen, Denmark; ^11^Psychiatric University Clinics Basel, Basel, Switzerland; ^12^Institute of Mental Health, Singapore, Singapore; ^13^School of Medicine and Lipid Research Centre, University of Wollongong, Wollongong, NSW, Australia; ^14^Illawarra Health and Medical Research Institute, University of Wollongong, Wollongong, NSW, Australia; ^15^Institute of Brain, Behaviour, and Mental Health, University of Manchester, Manchester, United Kingdom

**Keywords:** ultra-high risk, omega-3 fatty acids, psychosis, psychopathology, outcomes

## Abstract

**Background:**

Deficiencies in membrane polyunsaturated fatty acids (PUFA) such as omega-3 (n-3) fatty acids are thought to contribute to the pathophysiological processes underlying psychotic disorders. Emerging evidence suggests that the levels of PUFA are related to clinical symptoms but significant heterogeneity exists between studies. Here, we investigated associations of membrane PUFA with clinical symptoms and functioning in a large sample of individuals at ultra-high risk (UHR) for psychosis.

**Methods:**

A total of 285 participants of the NEURAPRO clinical trial were investigated for erythrocyte PUFA levels, including the n-3 index, n-6/n-3 PUFA ratio, docosahexaenoic acid (DHA), and eicosapentaenoic acid (EPA). Severity of general psychopathology [Brief Psychiatric Rating Scale (BPRS)], psychotic symptoms (BPRS psychosis subscale), negative symptoms [Scale for the Assessment of Negative Symptoms (SANS)], manic symptoms [Young Mania Rating Scale (YMRS)], depressive symptoms [Montgomery Asberg Depression Rating Scale (MADRS)], and functioning [Social and Occupational Functioning Scale (SOFAS), Global Functioning Social (GF-S) and Role (GF-R) scales] were assessed concurrently. Partial correlation taking into account the effects of gender, age, and smoking was used to examine the relationship between PUFAs and symptoms severity.

**Results:**

The n-3 index negatively correlated with the severity of general psychopathology, psychotic symptoms, depressive symptoms, and manic symptoms. The n-6/n-3 PUFA ratio positively correlated with severity of psychotic and depressive symptoms. The n-3 PUFA DHA negatively correlated with the severity of general psychopathology, positive, manic, and depressive symptoms. EPA negatively correlated with manic symptoms. Nervonic acid, an n-9 monounsaturated fatty acid, positively correlated with general psychopathology, positive and negative symptoms, depressive symptoms, and manic symptoms. The long-chain saturated fatty acid tetracosanoic acid positively correlated with general psychopathology, positive, manic, and depressive symptoms.

**Conclusions:**

Partially consistent with a previous study, psychotic symptoms, depressive symptoms, and symptoms of mania were associated with several classes of FAs in the present study. These findings support the relevance of membrane fatty acids for the onset of psychotic symptoms and indicate that FAs should be further evaluated as biomarkers in the UHR for psychosis group.

**Clinical Trial Registration:**

ANZCTR, identifier: 12608000475347

## Introduction

Young people at ultra-high risk (UHR) for psychosis have an elevated risk of developing psychosis of about 20% within 2 years and a significant risk for non-psychotic disorders (1, 2). The biological correlates underlying this risk state remain poorly understood but emerging evidence suggests the involvement of brain structural and functional alterations as well as peripheral pathology including immune activation, oxidative stress, endocrine abnormalities and deregulation of membrane lipid metabolism (3, 4). Given that it is currently unclear how to determine the biological risk for psychosis transition and other clinically relevant outcomes and how to develop treatments that target specific pathophysiological processes, it is important to elucidate the biological processes accompanying the UHR state. Moreover, declining transition rates in individuals who are clinically identified as being at UHR for psychosis (5) as well as uncertainty about the efficacy of specific interventions (6, 7) further add to the need to refine and improve risk prediction and consequently the identification of predictive biomarkers.

Deficits in long-chain omega-3 (n-3) polyunsaturated fatty acids (PUFAs) are one mechanism thought to contribute to psychosis risk (8, 9). In fact, n-3 PUFAs are integral components of the lipid bilayer forming the cell membrane and are found in high abundance in the human brain (10). There, they modulate the rigid formation of tightly packed saturated fatty acids through their structural characteristics, thereby also contributing to the biophysical properties of the neuronal membrane (11, 12). These in turn affect the function of ion channels and neurotransmitter receptors. Importantly, n-3 PUFA are essential fatty acids, meaning that their abundance in cell membranes is determined by intake from food and metabolic conversion. The lipid derivates of n-3 PUFA include anti-inflammatory eicosanoids such as resins and resolvins, which mediate the effects of n-3 PUFA on immune function (13, 14). Molecules derived from n-6 PUFA in contrast are known to have pro-inflammatory properties. First postulated by Horrobin et al. (15), the phospholipid structure of the cell membrane may be altered in schizophrenia and such alterations may contribute to various aspects of the pathophysiology observed in psychotic disorders, including neurotransmission, immune activation, and antioxidative defense. This is supported by studies showing n-3 PUFA deficiency in patients with schizophrenia compared to controls (16). Much less is known about the UHR for psychosis state, but evidence suggests lower levels of several n-3 and n-6 PUFA (17). Additionally, the balance between n-3 and n-6 PUFA, which is often seen as an indicator of immune activation, seems to be associated with depression in young people at UHR for psychosis (18).

More recently, clinical trials have tested the efficacy of n-3 PUFA for the prevention of psychosis transition in UHR individuals (19–21). While the first single-center trial showed significant benefits of n-3 PUFA over placebo in terms of the transition to psychosis risk (19), a multicenter replication study failed to show significant effects of n-3 PUFA supplementation on the transition rate (21). Possible reasons for the failure to replicate a reduction of transitions include true inefficacy of n-3 PUFA in this particular outcome measure, the background intervention of high-quality psychosocial care in both groups that may have created a ceiling effect, the use of antidepressants, or the high rate of non-adherence (57%) (22). Importantly, investigating the relationship between pre-treatment PUFA levels and symptom measures can provide further insight into potential mechanisms underlying lipid biology in regard to psychosis risk. A secondary analysis of the original single-center trial revealed associations of several classes of fatty acids with clinical symptoms and psychosocial functioning (23). In light of these observations, it seems important to replicate the findings of the original trial in the larger sample of the NEURAPRO clinical trial.

The aim of the present analysis was to examine associations of the cell membrane levels of fatty acids with clinical symptoms and functioning at baseline (i.e., prior to the intervention) in the NEURAPRO RCT. Specifically, we hypothesized that the n-3 index would be associated with less severe symptoms and better functioning. Given the limited evidence for fatty acid variations in the UHR stage, we did not formulate specific hypotheses for other fatty acid classes.

## Methods

### Participants

The study cohort consisted of 285 of 304 (94%) participants in the NEURAPRO study who provided consent for additional biomarker analysis. NEURAPRO was a double-blind placebo-controlled randomized clinical trial of fish oil (1.4 g fish oil/day) in people at UHR for psychosis (21) (ANZCTR identifier: 12608000475347) with 10 study sites (Amsterdam, Basel, Copenhagen, Jena, Hong Kong, Melbourne, Singapore, Sydney, Vienna, and Zurich). The intervention was administered in addition to cognitive behavioral case management (CBCM) for 6 months followed by a 6-month follow-up period. Help-seeking individuals attending UHR services in the trial centers were eligible if they were aged 13–40 years and met UHR criteria (24, 25). Exclusion criteria were a previous psychotic episode, acute intoxication, organic brain disease, serious developmental disorder, abnormal coagulation profile or thyroid function, physical illness with a psychotropic effect, current treatment with mood stabilizers, past neuroleptic exposure to a total lifetime haloperidol equivalent dose of more than 50 mg, IQ of less than 70, dangerous behavior, aggression or suicidality, pregnancy, or current supplementation with n-3 PUFA (25). All participants provided written informed consent (parent/guardian consent for participants aged <17 years). The NEURAPRO study was approved by the local human research ethics committees of the study centers.

### Data Collection

Psychiatric symptoms at baseline were assessed with the Brief Psychiatric Rating Scale (BPRS), the Scale for the Assessment of Negative Symptoms (SANS), the Montgomery Asberg Depression Rating Scale (MADRS) for depressive symptoms, the Young Mania Rating Scale (YMRS) for manic symptoms, the Social and Occupational Functioning Scale (SOFAS), and the Global Functioning Social (GF-S) and Role (GF-R) scales.

### Fatty Acid Analysis

Total fatty acid levels were quantified from erythrocyte samples collected at baseline. Erythrocytes were separated from plasma and extracted using an automated extraction method described previously by (26). Fatty acid levels were measured using mass spectrometry utilizing a hybrid triple quadrupole linear ion trap mass spectrometer (QTRAP 5500 AB Sciex, MA, USA) with an automated chip-based nanoelectrospray source (Triversa NanoMate, Advion Biosciences, New York, USA). Ionized lipids identified with a minimum signal-to-noise ratio of 10 were included in the analysis. Identification and quantification was accomplished using LipidView (v1.2, Sciex, MA, USA). Quantification was performed using LipidView software by comparing the spectral peak area of individual lipids to their class specific internal standards following isotope correction. Mass spectrometry is unable to identify fatty acid double bond isomers which limited our ability to distinguish between 18:1n-7/9, 20:3n-3/6/9 and 22:5n-3/6, respectively.

### Statistical Analysis

The n-3 index was calculated as the proportion of eicosapentaenoic acid (EPA) and docosahexaenoic acid (DHA) expressed as percent of total fatty acids. The n-6/n-3 PUFA ratio was calculated as arachidonic acid (AA) divided by the sum of EPA and DHA. To examine the relationship between PUFA and psychopathology, partial correlation coefficients (adj. R) were calculated adjusting for age, gender, and smoking, as these covariates have been found to influence both fatty acid levels and psychopathology. Given the explorative nature of the study and the fact that variations in PUFA concentrations in the UHR stage is still in a hypothesis-generating phase, we did not to correct for multiple testing. The significance for all tests was set at α < 0.05. Stata 13.1 (StataCorp, College Station, TX, USA) for Mac OS was used for all analyses.

## Results

Relevant demographic and psychological variables are presented **Table 1**. The sample comprised 285 participants (mean age 19.0 years ± 4.5; 45.26% male).

**Table 1:** Demographic and clinical characteristics and inflammatory markers of 285 NEURAPRO study participants.

Age, mean (SD)	18.97 (4.49)
Sex	
Female, n (%)	156 (54.74)
Male, n (%)	129 (45.26)
Smoking, n (%)	110 (38.6)
BPRS, mean (SD)	41.02 (9.72)
SANS, mean (SD)	17.79 (12.81)
MADRS, mean (SD)	19.27 (8.97)
SOFAS, mean (SD)	53.30 (12.44)
GF-S, mean (SD)	6.51 (1.22)
GF-R, mean (SD)	5.95 (1.54)

BPRS, Brief Psychiatric Rating Scale; MADRS, Montgomery-Asberg Depression Rating Scale; SANS, Scale for the Assessment of Negative Symptoms; SOFAS, Social and Occupational Functioning Scale.

Partial correlations between fatty acids and related indices and psychological variables, adjusted for age, gender and smoking, are reported in **Table 2**. BPRS scores were positively correlated with 24:0 (p < 0.001), 24:1n-9 (p < 0.001) and the n-6/3 PUFA ratio (p=0.01), and negatively correlated with the n-3 index (p=0.003), **20:3** (p=0.010), 22:6n-3 (p < 0.001), 20:4n-6 (p=0.037) and 18:2n-6 (p < 0.001). The BPRS psychotic symptoms sub-scale was positively correlated with 24:1n-9 (p=0.009) and with the n-6/3 PUFA ratio (p=0.01), and negatively correlated with 18:2n-6 (p=0.04) and the n-3 index (p=0.008). Negative symptoms (SANS) were positively correlated with 24:0 (p < 0.001) and 24:1n-9 (p < 0.001) and negatively correlated with 16:0 (p < 0.001), 20:3 (p < 0.001), 18:2n-6 (p < 0.001) and 18:1 (p=0.004).

**Table 2:** Partial correlations of fatty acids with symptom and functioning scores in 285 participants of the Neurapro-E study.

	BPRS		BPRS psy		SANS		MADRS		YMRS		SOFAS		GF-S		GF-R	
	adj. R	*p*-value	adj. R	*p*-value	adj. R	*p*-value	adj. R	*p*-value	adj. R	*p*-value	adj. R	*p*-value	adj. R	*p*-value	adj. R	*p*-value
16:0	-0.111	0.06	-0.011	0.86	**-0.210**	**<0.001**	-0.082	0.16	0.009	0.86	0.104	0.08	0.105	0.08	0.072	0.23
17:0	-0.015	0.79	0.029	0.63	-0.084	0.16	-0.011	0.85	-0.006	0.91	0.034	0.57	0.085	0.16	**0.136**	**0.02**
18:0	-0.029	0.62	-0.019	0.76	0.053	0.37	0.039	0.50	-0.055	0.35	**-0.129**	**0.03**	-0.113	0.06	-0.112	0.06
18:1*	**-0.119**	**0.04**	-0.008	0.90	**-0.191**	**0.001**	**-0.123**	**0.03**	**-0.140**	**0.02**	0.109	0.06	0.065	0.28	**0.121**	**0.04**
18:2n-6	**-0.344**	**<0.0001**	**-0.119**	**0.04**	**-0.328**	**<0.0001**	**-0.264**	**<0.0001**	**-0.324**	**<0.0001**	0.033	0.57	**0.146**	**0.01**	0.047	0.44
20:3#	**-0.175**	**0.003**	-0.105	0.08	**-0.202**	**<0.001**	**-0.192**	**0.001**	**-0.184**	**0.002**	0.102	0.08	**0.153**	**0.01**	**0.150**	**0.01**
20:4n-6	**-0.162**	**0.006**	-0.075	0.21	0.016	0.78	**-0.122**	**0.03**	**-0.280**	**<0.0001**	-0.016	0.78	0.016	0.79	-0.044	0.47
20:5n-3	0.005	0.92	-0.069	0.25	0.099	0.09	-0.039	0.50	**-0.128**	**0.03**	0.048	0.42	0.027	0.65	0.022	0.72
22:4n-6	-0.017	0.77	-0.065	0.28	0.109	0.07	-0.053	0.36	-0.080	0.18	-0.047	0.43	-0.071	0.24	**-0.129**	**0.03**
22:5^	0.015	0.80	0.040	0.50	0.113	0.06	0.082	0.16	**0.144**	**0.02**	-0.002	0.97	-0.067	0.27	-0.116	0.05
22:6n-3	**-0.198**	**<0.001**	**-0.166**	**0.006**	-0.081	0.17	**-0.191**	**0.001**	**-0.234**	**<0.0001**	0.026	0.65	-0.007	0.90	-0.053	0.38
24:0	**0.389**	**<0.001**	0.099	0.09	**0.289**	**<0.0001**	**0.307**	**<0.0001**	**0.375**	**<0.0001**	-0.074	0.21	-0.098	0.10	-0.005	0.93
24:1n-9	**0.365**	**<0.001**	**0.157**	**0.009**	**0.293**	**<0.0001**	**0.307**	**<0.0001**	**0.377**	**<0.0001**	-0.074	0.21	-0.115	0.05	-0.047	0.44
n-3 index^†^	**-0.176**	**0.003**	**-0.160**	**0.008**	-0.057	0.34	**-0.177**	**0.002**	**-0.230**	**<0.0001**	0.031	0.59	-0.002	0.97	-0.044	0.47
n-6/3 ratio^&^	**0.153**	**0.01**	**0.147**	**0.01**	0.093	0.12	**0.169**	**0.005**	0.118	0.05	-0.083	0.16	-0.021	0.73	0.035	0.56

* 18:1 could be 18:1n-7 or 18:1n-9, or a combination of both.

# 20:3 could be 20:3n-3, 20:3n-6 or 20:3n-9, or a combination of all 3 isomers.

^ 22:5 could be 22:5n-3 or 22:5n-6, or a combination of both.

^†^The n-3 index is calculated as the amount of EPA and DHA expressed as the percent of total fatty acids.

^&^The n-6/3 ratio is calculated as AA/(EPA + DHA).

Bold numbers indicate statistical significance.

YMRS scores were positively correlated with 24:0 (p < 0.0001) and 24:1n-9 (p < 0.0001) and negatively correlated with 20:3 (p=0.002),20:5n-3 (p=0.03), 22:6n-3 (p < 0.0001), 18:2n-6 (p < 0.0011), 20:4n-6 (p < 0.0001) and the n-3-index (p < 0.0001). MADRS scores were positively correlated with 24:0 (p < 0.0001), 24:1n-9 (p < 0.0001) and the n-6/3 PUFA ratio (p=0.005), and negatively correlated with the n-3-index (p=0.002), 20:3 (p=0.001), 22:6n-3 (p=0.001), 18:2n-6 (p < 0.0001), 20:4n-6 (p=0.03) and 18:1 (p=0.03). SOFAS scores were negatively correlated with 18:0 (p=0.031). GF-S scores were positively correlated with 18:2n-6 (p < 0.01) and 20:3 (p=0.01). Finally, GF-R scores were positively correlated with 17:0 (p=0.02), 18:1 (p=0.04), 22:4n-6 (p=0.03) and 20:3 (p=0.01).

## Discussion

The aim of this study was to investigate associations of cell membrane fatty acids with clinical characteristics in a large multi-centre RCT of fish oil supplementation in individuals at UHR for psychosis. After taking into account the effects of age, gender and smoking, we found several PUFAs related to psychopathology, including general psychopathology (BPRS), psychotic symptoms (BPRS), negative symptoms (SANS), manic symptoms (YMRS) and depressive symptoms (MADRS). The n-3 index, DHA, AA, 20:3, and 18:2n6 were negatively correlated with BPRS, MADRS and YMRS scores, while the n-6/3 PUFA ratio was positively correlated with general psychopathology (BPRS), psychotic symptoms (BPRS) and depressive symptoms (MADRS). 24:0 and 24:1n-9 in turn were significantly positively correlated with BPRS, SANS, MADRS and YMRS scores. Collectively, these findings support the notion that several classes of fatty acids are associated with symptom severity in this group.

Our analysis contributes important insights into the relationship between PUFA and psychopathology by replicating findings of our previous smaller study in UHR (23) in a large (n=285) and well-characterised sample. Overall, our results confirm that higher levels of n-3 PUFA (n-3 index) correspond to fewer symptoms in this population. The n-3 index, calculated as the sum of EPA and DHA as percent of total fatty acids, was significantly associated with lower general psychopathology (BPRS) scores, which is broadly consistent with a previous study (27), as well as with lower manic (YMRS) and depressive (MADRS) symptoms. The n-6/n-3 PUFA ratio, reflective of the balance between n-3 and n-6 PUFA, was positively correlated with psychotic symptoms (BPRS) and MADRS scores, suggesting that a higher relative proportion of n-6 PUFA compared to n-3 PUFA correlates with more severe symptoms. Consistent with the present findings, Kim et al. (23) found that the n-6/n-3 PUFA ratio was associated with psychotic symptoms, and the sum of n-3 PUFA negatively with negative symptoms. This suggests that the n-3 index is relevant to a range of symptoms in the UHR state. Consistent with this, DHA was also associated with lower BPRS scores in our study. DHA is the most abundant n-3 PUFA in the human brain and integral to the neuronal membrane. Importantly, erythrocyte DHA correlates with grey matter DHA (28) and may be particularly relevant during adolescence, where increases in the level of DHA may be critical for the development of the prefrontal cortex and cortical maturation more generally (29–31). Given that the UHR state and the onset of psychotic disorders fall within this developmental period in many cases, n-3 PUFA including DHA may play a particularly important role in this phase.

Our results also support an association of the n-3 index and the n-6/n-3 PUFA ratio with depressive symptoms in this UHR cohort. Considering that young people at UHR for psychosis have a high risk for depression of up to 42% (1), these findings are highly relevant to the UHR group. There is ample evidence to support n-3 PUFA deficiency in patients with major depressive disorders (32) and for the efficacy of n-3 PUFA as a potential treatment of depressive symptoms [see meta-analyses in Refs. (33–35)]. Moreover, we recently reported that in individuals at UHR for psychosis, a high n-6/n-3 PUFA ratio is predictive of depression within a 7-year follow-up (18). Our present findings support an association of the n-3 index as well as of DHA levels with depressive symptoms, thereby confirming previous results. However, EPA, the n-3 PUFA found to be most effective for the treatment of depression (34), was not related to depressive symptoms in this study.

In addition to n-3 PUFA, linoleic acid (18:2), an n-6 PUFA and precursor to arachidonic acid, EPA and DHA, was inversely associated with symptoms severity and positively associated with functioning. Previous studies examining the relationship between linoleic acid and psychopathology have yielded mixed results. For example, in a recent study of 154 patients with major depressive disorder, linoleic acid was found to be reduced compared to controls and inversely correlated with depressive symptoms severity (36). Similarly, in a population-based study of otherwise healthy adults, low levels of linoleic acid were associated with depressive symptoms (37). However, other studies found no association of linoleic acid with depression (38, 39). While our results suggest that linoleic acid is associated with fewer symptoms and better functioning, these observations warrant further confirmation.

An interesting observation in our study was that tetracosanoic acid (lignoceric acid; 24:0), a very long chain saturated fatty acid mainly found in peanut and canola oil (40), was strongly positively correlated with all symptom scores. Few studies to date have investigated saturated fatty acids in the UHR group. Of note, Hamazaki et al. (41) found elevated levels of these fatty acids in post-mortem brain tissue of patients with schizophrenia compared to controls.

The mechanisms linking deficits in n-3 PUFA with psychopathology in the UHR group are only partially understood but likely include effects on serotonergic and dopaminergic neurotransmission through the modulation of membrane fluidity and ion channel function (42), PUFA effects on HPA axis regulation and the regulation of antioxidative defense (43), as well as the production of pro- and anti-inflammatory derivatives of PUFA (14, 44, 45). EPA rapidly beta-oxidized once in the brain (46, 47), and oxidation products are not specific for EPA. Therefore, EPA effects in depression cannot be explained by effects in the brain; rather, peripheral anti-inflammatory effects may be relevant. EPA’s oxidation products are anti-inflammatory eicosanoids, and oxidative stress as seen in psychiatric disorders would only increase these. This is supported by Rapaport et al. (48), who showed that high inflammation is a predictor of response to n-3 PUFA supplementation.

Other fatty acids related to psychopathology in our study include NA. NA, an n-9 monounsaturated fatty acid, is abundant in the white matter of the central nervous system and important for the biosynthesis and maintenance of the myelin sheath. For example, the levels of NA (but also AA and DHA) were found to be related to decreased white matter integrity in the corpus callosum, parietal, occipital, temporal, and frontal lobes (49). Impaired myelin pathways and impairments in white matter integrity in the medial frontal lobe and other brain areas relevant to psychosis have been observed in individuals who later transitioned to psychosis (50). Decreased levels of NA have previously been observed in association with more severe psychotic symptoms and with higher transition risk (23, 51). The present findings seem difficult to reconcile as they suggest the opposite relationship. However, differences in the methodology between the present study and previous studies may explain these discrepancies (52). While Amminger et al. (51) measured NA from the phosphatidylethanolamine fraction of erythrocytes using gas chromatographic analysis, NA was quantified in this study from whole erythrocyte membranes using mass spectrometry. In patients with major depressive disorder, where white matter dysfunction is similarly hypothesized, both increases and decreases in NA have been observed (52, 53).

An important question not answered by our study is whether young individuals at UHR for psychosis show deficits in those n-3 PUFAs associated with more severe symptoms compared to healthy (non-UHR) individuals. To date, only one study of our group addressed this question and reported deficits in two n-3 PUFAs and several n-6 PUFAs (17). We are currently investigating this question in the NEURAPRO trial and a matched control group. Studies in patients with chronic schizophrenia provided heterogeneous findings and showed decreases in AA, DPA, and DHA (54). However, several studies in schizophrenia patients including a more recent study showed increases in these fatty acids in patients compared to controls as well as in unaffected siblings compared to controls (55–57). Findings in youth at UHR for bipolar disorder showed deficits in the n-3-index compared to healthy controls (58, 59). It therefore seems important to clarify if low n-3 PUFA levels precede the onset of psychosis.

A strength of our study is the sample size, making this the largest examination of membrane PUFA in UHR to date. A second strength is the well-characterized sample and the availability of a variety of clinical measures. Limitations include the cross-sectional nature of this analysis, which precludes causal inferences. The effects sizes of several correlations reported here—while statistically significant—are small (adj. R < 0.2). Also, the selection of fatty acids measured is not fully consistent with a previous study (23) due to the choice of methods used for the analysis. The present study used mass spectrometry, a novel method recently published (26), while previous studies used mostly gas chromatography. While dietary habits were not considered here, we argue that membrane PUFA status is a valid biological measure of lipid biology irrespective of source. Finally, we did not correct for multiple comparisons in the present study due to its exploratory nature.

Future studies should further confirm if the UHR for psychosis group has deficits in PUFA compared to healthy individuals as well as other high-risk groups; clarify causality by testing whether the relationship with clinical symptoms persists at follow-up and is predictive of treatment response and long-term remission; if supplementation with fish oil can reverse potential deficits and associations with psychopathology; and whether the relationship between lipid biology and symptoms is attributable to inflammation or other biological mechanisms that may serve as potential treatment targets.

In conclusion, our study confirms that membrane PUFAs are associated with the severity of clinical symptoms in individuals at UHR for psychosis. In particular, we found that the n-3 index was negatively correlated with multiple measures, including total BPRS scores, MADRS, and YMRS scores. These results suggest that n-3 PUFA are important in the pathophysiology of the UHR state and may indicate risk for poor mental health outcomes. Given that recent RCTs of n-3 PUFA in the UHR were unable to demonstrate superiority of n-3 PUFA over placebo for transition to psychosis, biomarker studies are warranted to clarify if low n-3 PUFA levels predispose for adverse outcomes and are indicative of treatment response with n-3 PUFA. 

## Ethics Statement

This study was approved by the local human ethics review board of each site (Melbourne, Australia: Melbourne Health Research Ethics Committee; Sydney, Australia: Sydney South West Area Health Service Ethics Review Committee; Basel, Switzerland: Ethics Commission for Basel; Zurich, Switzerland: Cantonal Ethics Commission Zurich; Jena, Germany: University Clinic Jena Ethics Commission; Copenhagen, Denmark: Capital Region Research Ethics Committee; Hong Kong: Institutional Review Board of the University of Hong Kong/Hospital Authority Hong Kong West Cluster; Vienna, Austria: Medical University of Vienna Ethics Commission; Singapore: National Healthcare Group Domain Specific Review Board; and Amsterdam, the Netherlands: Academic Medical Centre Medical Ethics Committee). This study was carried out in accordance with the Declaration of Helsinki and The National Health and Medical Research Council of Australia National Statement on Human Research with written informed consent from all subjects. All subjects gave written informed consent in accordance with the Declaration of Helsinki.

## Author Contributions

MB and GA designed the study. MB analyzed the data and wrote the manuscript. BN, CM, HY, MRS, NM, MS, SS, IH, GB, EC, LH, DN, MN, AR-R, SV, AY, PM, and GA contributed to the primary study that provided data for this analysis, including acquisition of funding, recruitment of participants, and/or collection of data. TM and BM analyzed erythrocyte fatty acids. All authors contributed to the interpretation of results and to the manuscript.

## Funding

The NEURAPRO clinical trial was supported by grant 07TGF-1102 from the Stanley Medical Research Institute, grant 566529 from the NHMRC Australia, and a grant from the Colonial Foundation. The analysis of fatty acids was supported by grant 1128631 from the NHMRC Australia. MB is supported by a MACH Translational Research Fellowship. GB is supported by the Swiss National Science Foundation (SNF 33IC30_166826).

## Conflict of Interest Statement

The authors declare that the research was conducted in the absence of any commercial or financial relationships that could be construed as a potential conflict of interest.
