# Corrigendum: Relationship Between Polyunsaturated Fatty Acids and Psychopathology in the NEURAPRO Clinical Trial

**DOI:** 10.3389/fpsyt.2020.00514

**Published:** 2020-06-10

**Authors:** Maximus Berger, Barnaby Nelson, Connie Markulev, Hok Pan Yuen, Miriam R. Schäfer, Nilufar Mossaheb, Monika Schlögelhofer, Stefan Smesny, Ian B. Hickie, Gregor E. Berger, Eric Y. H. Chen, Lieuwe de Haan, Dorien H. Nieman, Merete Nordentoft, Anita Riecher-Rössler, Swapna Verma, Todd W. Mitchell, Barbara J. Meyer, Andrew Thompson, Alison Ruth Yung, Patrick D. McGorry, G. Paul Amminger

**Affiliations:** ^1^Orygen—The National Centre of Excellence in Youth Mental Health, Melbourne, VIC, Australia; ^2^Centre for Youth Mental Health, The University of Melbourne, Melbourne, VIC, Australia; ^3^Department of Psychiatry and Psychotherapy, Clinical Division of Social Psychiatry, Medical University Vienna, Vienna, Austria; ^4^Department of Child and Adolescent Psychiatry, Medical University Vienna, Vienna, Austria; ^5^Department of Psychiatry, University Hospital Jena, Jena, Germany; ^6^Brain and Mind Research Institute, University of Sydney, Sydney, NSW, Australia; ^7^Department of Child and Adolescent Psychiatry and Psychotherapy, University Hospital of Psychiatry Zurich, University of Zurich, Zurich, Switzerland; ^8^Department of Psychiatry, University of Hong Kong, Hong Kong, Hong Kong; ^9^Department of Psychiatry, Academic Medical Center, Amsterdam, Netherlands; ^10^Psychiatric Centre Bispebjerg, Copenhagen, Denmark; ^11^Psychiatric University Clinics Basel, Basel, Switzerland; ^12^Institute of Mental Health, Singapore, Singapore; ^13^School of Medicine and Lipid Research Centre, University of Wollongong, Wollongong, NSW, Australia; ^14^Illawarra Health and Medical Research Institute, University of Wollongong, Wollongong, NSW, Australia; ^15^Institute of Brain, Behaviour, and Mental Health, University of Manchester, Manchester, United Kingdom

**Keywords:** ultra-high risk, omega-3 fatty acids, psychosis, psychopathology, outcomes

In the original article, Todd W. Mitchell and Barbara J. Meyer were not included as authors. The corrected Author Contributions Statement appears below.

## Author Contributions

MB and GA designed the study. MB analyzed the data and wrote the manuscript. BN, CM, HY, MRS, NM, MS, SS, IH, GB, EC, LH, DN, MN, AR-R, SV, AY, PM, and GA contributed to the primary study that provided data for this analysis, including acquisition of funding, recruitment of participants, and/or collection of data. TM and BM analyzed erythrocyte fatty acids. All authors contributed to the interpretation of results and to the manuscript.

In addition, there was a mistake in **Table 2** as published. The names of the following fatty acids are incorrect: 18:1n-7, 20:3n-6 and 22:5n-3/6. They should be 18:1, 20:3 and 22:5, respectively. The corrected **Table 2** appears below and the relevant changes to the text have also been made.

**Table 2:** Partial correlations of fatty acids with symptom and functioning scores in 285 participants of the Neurapro-E study.

	BPRS		BPRS psy		SANS		MADRS		YMRS		SOFAS		GF-S		GF-R	
	adj. R	*p*-value	adj. R	*p*-value	adj. R	*p*-value	adj. R	*p*-value	adj. R	*p*-value	adj. R	*p*-value	adj. R	*p*-value	adj. R	*p*-value
16:0	-0.111	0.06	-0.011	0.86	**-0.210**	**<0.001**	-0.082	0.16	0.009	0.86	0.104	0.08	0.105	0.08	0.072	0.23
17:0	-0.015	0.79	0.029	0.63	-0.084	0.16	-0.011	0.85	-0.006	0.91	0.034	0.57	0.085	0.16	**0.136**	**0.02**
18:0	-0.029	0.62	-0.019	0.76	0.053	0.37	0.039	0.50	-0.055	0.35	**-0.129**	**0.03**	-0.113	0.06	-0.112	0.06
18:1*	**-0.119**	**0.04**	-0.008	0.90	**-0.191**	**0.001**	**-0.123**	**0.03**	**-0.140**	**0.02**	0.109	0.06	0.065	0.28	**0.121**	**0.04**
18:2n-6	**-0.344**	**<0.0001**	**-0.119**	**0.04**	**-0.328**	**<0.0001**	**-0.264**	**<0.0001**	**-0.324**	**<0.0001**	0.033	0.57	**0.146**	**0.01**	0.047	0.44
20:3#	**-0.175**	**0.003**	-0.105	0.08	**-0.202**	**<0.001**	**-0.192**	**0.001**	**-0.184**	**0.002**	0.102	0.08	**0.153**	**0.01**	**0.150**	**0.01**
20:4n-6	**-0.162**	**0.006**	-0.075	0.21	0.016	0.78	**-0.122**	**0.03**	**-0.280**	**<0.0001**	-0.016	0.78	0.016	0.79	-0.044	0.47
20:5n-3	0.005	0.92	-0.069	0.25	0.099	0.09	-0.039	0.50	**-0.128**	**0.03**	0.048	0.42	0.027	0.65	0.022	0.72
22:4n-6	-0.017	0.77	-0.065	0.28	0.109	0.07	-0.053	0.36	-0.080	0.18	-0.047	0.43	-0.071	0.24	**-0.129**	**0.03**
22:5^	0.015	0.80	0.040	0.50	0.113	0.06	0.082	0.16	**0.144**	**0.02**	-0.002	0.97	-0.067	0.27	-0.116	0.05
22:6n-3	**-0.198**	**<0.001**	**-0.166**	**0.006**	-0.081	0.17	**-0.191**	**0.001**	**-0.234**	**<0.0001**	0.026	0.65	-0.007	0.90	-0.053	0.38
24:0	**0.389**	**<0.001**	0.099	0.09	**0.289**	**<0.0001**	**0.307**	**<0.0001**	**0.375**	**<0.0001**	-0.074	0.21	-0.098	0.10	-0.005	0.93
24:1n-9	**0.365**	**<0.001**	**0.157**	**0.009**	**0.293**	**<0.0001**	**0.307**	**<0.0001**	**0.377**	**<0.0001**	-0.074	0.21	-0.115	0.05	-0.047	0.44
n-3 index^†^	**-0.176**	**0.003**	**-0.160**	**0.008**	-0.057	0.34	**-0.177**	**0.002**	**-0.230**	**<0.0001**	0.031	0.59	-0.002	0.97	-0.044	0.47
n-6/3 ratio^&^	**0.153**	**0.01**	**0.147**	**0.01**	0.093	0.12	**0.169**	**0.005**	0.118	0.05	-0.083	0.16	-0.021	0.73	0.035	0.56

* 18:1 could be 18:1n-7 or 18:1n-9, or a combination of both.

# 20:3 could be 20:3n-3, 20:3n-6 or 20:3n-9, or a combination of all 3 isomers.

^ 22:5 could be 22:5n-3 or 22:5n-6, or a combination of both.

^†^The n-3 index is calculated as the amount of EPA and DHA expressed as the percent of total fatty acids.

^&^The n-6/3 ratio is calculated as AA/(EPA + DHA).

Bold numbers indicate statistical significance.

The *Methods* section, subsection *Fatty Acid Analysis*:

“Total fatty acid levels were quantified from erythrocyte samples collected at baseline. Erythrocytes were separated from plasma and extracted using an automated extraction method described previously by (26). Fatty acid levels were measured using mass spectrometry utilizing a hybrid triple quadrupole linear ion trap mass spectrometer (QTRAP 5500 AB Sciex, MA, USA) with an automated chip-based nanoelectrospray source (Triversa NanoMate, Advion Biosciences, New York, USA). Ionized lipids identified with a minimum signal-to-noise ratio of 10 were included in the analysis. Identification and quantification was accomplished using LipidView (v1.2, Sciex, MA, USA). Quantification was performed using LipidView software by comparing the spectral peak area of individual lipids to their class specific internal standards following isotope correction. Mass spectrometry is unable to identify fatty acid double bond isomers which limited our ability to distinguish between 18:1n-7/9, 20:3n-3/6/9 and 22:5n-3/6, respectively.”

A correction has been made to *Results* section, paragraphs 2 and 3:

“Partial correlations between fatty acids and related indices and psychological variables, adjusted for age, gender and smoking, are reported in **Table 2**. BPRS scores were positively correlated with 24:0 (p < 0.001), 24:1n-9 (p < 0.001) and the n-6/3 PUFA ratio (p=0.01), and negatively correlated with the n-3 index (p=0.003), **20:3** (p=0.010), 22:6n-3 (p < 0.001), 20:4n-6 (p=0.037) and 18:2n-6 (p < 0.001). The BPRS psychotic symptoms sub-scale was positively correlated with 24:1n-9 (p=0.009) and with the n-6/3 PUFA ratio (p=0.01), and negatively correlated with 18:2n-6 (p=0.04) and the n-3 index (p=0.008). Negative symptoms (SANS) were positively correlated with 24:0 (p < 0.001) and 24:1n-9 (p < 0.001) and negatively correlated with 16:0 (p < 0.001), 20:3 (p < 0.001), 18:2n-6 (p < 0.001) and 18:1 (p=0.004).”

“YMRS scores were positively correlated with 24:0 (p < 0.0001) and 24:1n-9 (p < 0.0001) and negatively correlated with 20:3 (p=0.002),20:5n-3 (p=0.03), 22:6n-3 (p < 0.0001), 18:2n-6 (p < 0.0011), 20:4n-6 (p < 0.0001) and the n-3-index (p < 0.0001). MADRS scores were positively correlated with 24:0 (p < 0.0001), 24:1n-9 (p < 0.0001) and the n-6/3 PUFA ratio (p=0.005), and negatively correlated with the n-3-index (p=0.002), 20:3 (p=0.001), 22:6n-3 (p=0.001), 18:2n-6 (p < 0.0001), 20:4n-6 (p=0.03) and 18:1 (p=0.03). SOFAS scores were negatively correlated with 18:0 (p=0.031). GF-S scores were positively correlated with 18:2n-6 (p < 0.01) and 20:3 (p=0.01). Finally, GF-R scores were positively correlated with 17:0 (p=0.02), 18:1 (p=0.04), 22:4n-6 (p=0.03) and 20:3 (p=0.01).”

A correction has also been made to the *Discussion* section, paragraph 1:

“The aim of this study was to investigate associations of cell membrane fatty acids with clinical characteristics in a large multi-centre RCT of fish oil supplementation in individuals at UHR for psychosis. After taking into account the effects of age, gender and smoking, we found several PUFAs related to psychopathology, including general psychopathology (BPRS), psychotic symptoms (BPRS), negative symptoms (SANS), manic symptoms (YMRS) and depressive symptoms (MADRS). The n-3 index, DHA, AA, 20:3, and 18:2n6 were negatively correlated with BPRS, MADRS and YMRS scores, while the n-6/3 PUFA ratio was positively correlated with general psychopathology (BPRS), psychotic symptoms (BPRS) and depressive symptoms (MADRS). 24:0 and 24:1n-9 in turn were significantly positively correlated with BPRS, SANS, MADRS and YMRS scores. Collectively, these findings support the notion that several classes of fatty acids are associated with symptom severity in this group.”

Finally, the n-3 index was incorrectly described as the proportion of eicosapentaenoic acid (EPA) and docosahexaenoic acid (DHA) relative to the total amount of n-3 fatty acids. It should be “The n-3 index was calculated as the proportion of eicosapentaenoic acid (EPA) and docosahexaenoic acid (DHA) expressed as percent of total fatty acids.”

A correction has been made to *Methods*, subsection *Statistical Analysis*:

“The n-3 index has been calculated as the proportion of eicosapentaenoic acid (EPA) and docosahexaenoic acid (DHA) relative to the total amount of n-3 fatty acids” has been changed to “The n-3 index was calculated as the proportion of eicosapentaenoic acid (EPA) and docosahexaenoic acid (DHA) expressed as percent of total fatty acids.”

A correction has been made to the *Discussion* section, paragraph 2:

“Our analysis contributes important insights into the relationship between PUFA and psychopathology by replicating findings of our previous smaller study in UHR (23) in a large (n=285) and well-characterised sample. Overall, our results confirm that higher levels of n-3 PUFA (n-3 index) correspond to fewer symptoms in this population. The n-3 index, calculated as the sum of EPA and DHA as percent of total fatty acids, was significantly associated with lower general psychopathology (BPRS) scores, which is broadly consistent with a previous study (27), as well as with lower manic (YMRS) and depressive (MADRS) symptoms. The n-6/n-3 PUFA ratio, reflective of the balance between n-3 and n-6 PUFA, was positively correlated with psychotic symptoms (BPRS) and MADRS scores, suggesting that a higher relative proportion of n-6 PUFA compared to n-3 PUFA correlates with more severe symptoms. Consistent with the present findings, Kim et al. (23) found that the n-6/n-3 PUFA ratio was associated with psychotic symptoms, and the sum of n-3 PUFA negatively with negative symptoms. This suggests that the n-3 index is relevant to a range of symptoms in the UHR state. Consistent with this, DHA was also associated with lower BPRS scores in our study. DHA is the most abundant n-3 PUFA in the human brain and integral to the neuronal membrane. Importantly, erythrocyte DHA correlates with grey matter DHA (28) and may be particularly relevant during adolescence, where increases in the level of DHA may be critical for the development of the prefrontal cortex and cortical maturation more generally (29–31). Given that the UHR state and the onset of psychotic disorders fall within this developmental period in many cases, n-3 PUFA including DHA may play a particularly important role in this phase.”

The authors apologize for these errors and state that they do not change the scientific conclusions of the article in any way. The original article has been updated.

